# A Unique Case of Fungal Endometritis Caused by *Aspergillus quadrilineatus* in an Immunocompetent Woman and Literature Review

**DOI:** 10.1002/ccr3.70146

**Published:** 2025-02-12

**Authors:** Fatemeh Zahra Ranjbar Golafshani, Erfan Ghaffari Lashkenari, Firoozeh Kermani, Soheila Abbaszadeh Godarzi, Saeid Mahdavi Omran

**Affiliations:** ^1^ Parasitology and medical mycology Department, Faculty of Medicine Babol University of Medical Sciences Babol Iran; ^2^ Infectious Diseases and Tropical Medicine Research Center Health Research Institute, Babol University of Medical Sciences Babol Iran; ^3^ Student Research Committee Babol University of Medical Sciences Babol Iran; ^4^ Obstetrics and Gynecology Department, Faculty of Medicine Babol University of Medical Sciences Babol Iran; ^5^ Infertility and Reproductive Health Research Center Health Research Institute, Rouhani Hospital, Babol University of Medical Sciences Babol Iran

**Keywords:** *Aspergillus quadrilineatus*, endometrial aspergillosis, endometritis, metrorrhagia

## Abstract

Abnormal uterine bleeding (AUB) is a common gynecological concern often attributed to hormonal disorders, malignancies, or infections. While bacterial and viral infections are frequently investigated, fungal infections, such as aspergillosis, are often overlooked. This case report highlights a rare instance of endometrial aspergillosis caused by *Aspergillus quadrilineatus* in an immunocompetent woman. A 64‐year‐old woman presented with persistent vaginal bleeding. Histopathological examination of endometrial tissue revealed septate hyphae consistent with fungal infection. Molecular analysis identified the causative agent as *A. quadrilineatus* . The *A. quadrilineatus* isolate demonstrated susceptibility to various antifungal agents, including azoles and amphotericin B. This report emphasizes the need for increased awareness of fungal infections, including aspergillosis, as a potential cause of AUB. Further research is needed to enhance understanding of the epidemiology, risk factors, and clinical manifestations of endometrial aspergillosis.

**Summary**

- Endometrial aspergillosis is an uncommon yet potentially life‐threatening condition.
- In patients presenting with abnormal uterine bleeding and elevated inflammatory markers, an endometrial biopsy should be considered to exclude fungal infections.
- Timely diagnosis and prompt intervention with antifungal therapy and surgical debridement are essential for optimal outcomes.

## 1 Introduction

Patients with abnormal uterine bleeding (AUB) are primarily assessed for hormonal disorders, malignancies, cysts, myomas, and infections [1]. Current investigations predominantly target bacterial and viral infections, while fungal infections are rarely addressed in humans [2]. Fungi have the capacity to induce reproductive failure in animals either by directly establishing infections within the reproductive system or by producing toxic metabolites in vitro, which are subsequently absorbed by the animal [3]. Fungi have been documented in various pathological conditions of the genital tract, including cervicitis, vulvovaginitis, abortion, and endometritis [4]. Aspergillosis poses significant morbidity and accounts for over one million fatalities annually among immunocompromised individuals globally [5]. *Aspergillus* is a genus of filamentous fungi commonly found in the environment. It produces a wide variety of conidia (asexual spores) that can be easily inhaled, leading to various human infections, ranging from allergic bronchopulmonary aspergillosis (ABPA) and endocarditis to life‐threatening invasive aspergillosis [6]. The diverse and often severe clinical manifestations complicate diagnosis and management [2]. The diagnosis of these infections can be challenging and requires a comprehensive approach that includes clinical, radiological, and mycological findings. Traditional diagnostic methods encompass microscopy, culture, and serological assays, while molecular techniques such as polymerase chain reaction (PCR) and sequencing offer improved sensitivity and specificity, especially in cases of invasive disease [5]. Co‐infection with *Aspergillus* species and squamous cell carcinoma of the cervix in immunocompromised women is frequently associated with underlying malignancy [7]. Additionally, previous research has indicated the impact of fungal toxins on the ovary and uterus [8]. Clinical manifestations of the disease are heterogeneous and vary according to the severity of the condition, the level of immune compromise, and the nature and intensity of the host's immune response [9]. Endometrial aspergillosis presents considerable diagnostic challenges due to its rarity and nonspecific clinical manifestations. The overlap of symptoms with other gynecological infections, such as bacterial vaginitis and candidiasis, often contributes to misdiagnosis [10]. Furthermore, the low prevalence of *Aspergillus* infections within the female genital tract results in a limited number of clinical cases available for study and the formulation of diagnostic algorithms [11]. The absence of a gold standard diagnostic test, coupled with the potential for asymptomatic colonization, exacerbates the difficulties associated with achieving a definitive diagnosis. Consequently, endometrial aspergillosis is often underdiagnosed, which can lead to delayed or inappropriate treatment and may result in severe complications [12].

This case presents a unique instance of endometrial *Aspergillus* infection attributed to *A. quadrilineatus* in a woman with AUB. The objective is to highlight an unexpected site of *Aspergillus* infection; to the best of our knowledge, this is the first recorded case of isolated endometrial aspergillosis in an immunocompetent patient.

## 2 Case History/Examination

A 64‐year‐old female with no previous medical history was admitted to the emergency department of a Medical Center in Babol, north of Iran on April 26, 2024. The patient, with a history of two normal vaginal births and 15 years of menopause, was referred for persistent vaginal bleeding over the past 3 months without any medical intervention.

**FIGURE 1:**
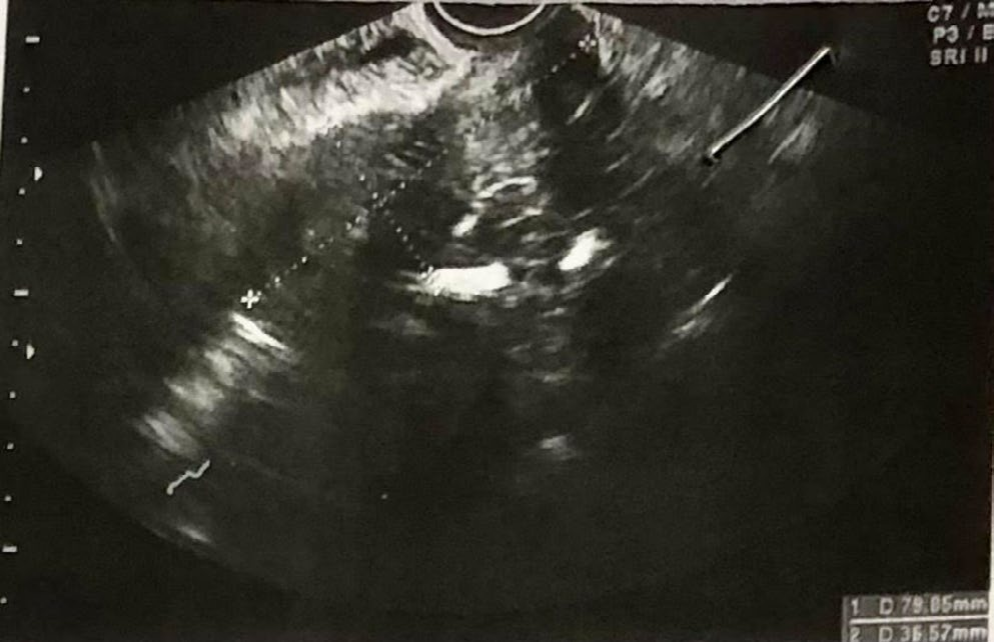
Ultrasound examination of the uterus and adnexa (abdominal).

A vaginal examination conducted by a gynecologist revealed no evidence of any wounds or infections in the patient. Examination revealed vital signs: blood pressure 120/90 mmHg, temperature 36.3°C, respiratory rate 18 breaths/min, and pulse rate 80 beats/min. There was no reported history of underlying diseases or previous surgeries. Notably, the patient's medical history indicated the onset of lung disease 2 months prior, for which she was using salbutamol 2–4 mg tablet, 3–4 times daily for management. The hematological work‐up showed: white blood cell count; 4.53 × 10^3^/μl (neutrophils; 49%, lymphocytes; 37% and monocytes; 8.3%), hemoglobin; 12.7 mg/dL, RBC count; 4.29 × 10^6^/μ, hematocrit; 38.7% and platelet count; 236 × 10^3^/μL. The patient's thyroid‐stimulating hormone (TSH) and prolactin levels were within the normal range and the beta‐human chorionic gonadotropin (BHCG) test indicated a negative result of 0.3. Ultrasound examination of the uterus and adnexa (abdominal and vaginal) was performed. The uterus measured 58 × 32 mm in the axial view, with uniform myometrium echogenicity. The endometrial thickness was 9.7 mm, above the normal range, exhibiting a heterogeneous texture with micro cystic foci; correlation with histopathological findings is recommended. An accumulation of fluid measuring 4.5 mm in thickness was noted in the inferior endometrial cavity. Ovarian dimensions were recorded as 22 × 10 mm, appearing atrophic, with no cystic or solid lesions in the adnexa. No free fluid was detected in the pelvic cavity (Figure 1). It is noteworthy that the patient has experienced a weight loss of 4 kg over a period of 2 months. Based on the examination results, the patient has qualified as a candidate for fractional dilation and curettage (D&C).

**FIGURE 2:**
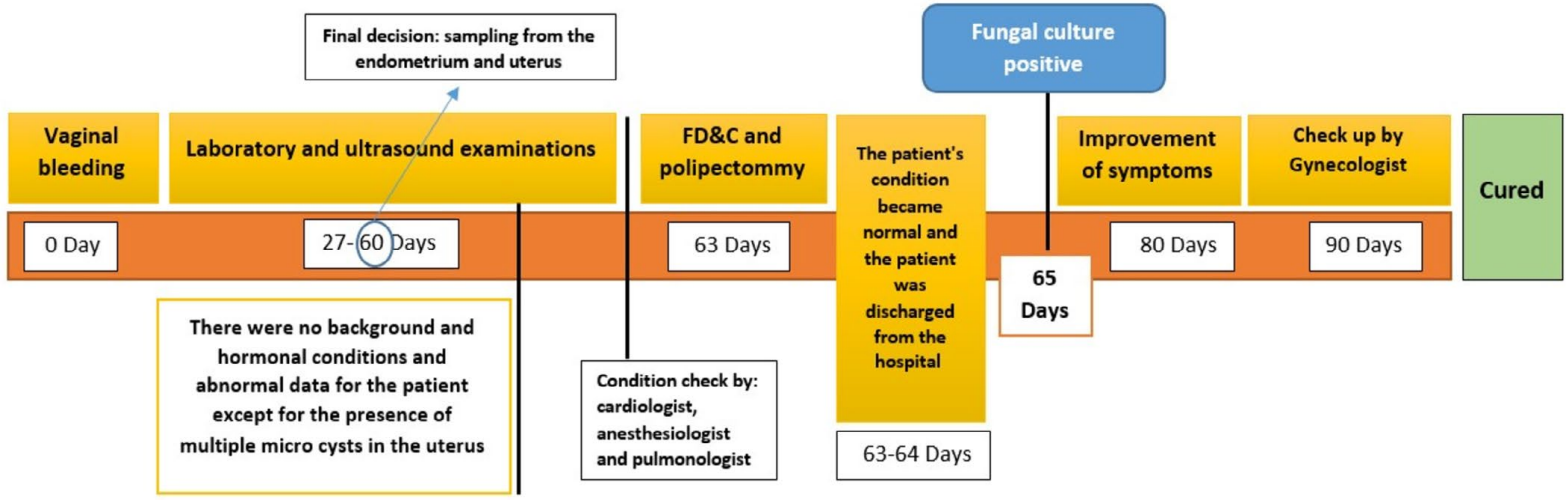
Timeline of disease progression in the course of endometritis with abnormal uterine bleeding, and proven aspergillosis caused by *Aspergillus quadrilineatus*.

The patient was reexamined, and based on fever, vaginal bleeding, and pain; the urine analysis sample was collected and showed few bacteria. The patient underwent uterine sampling. The vagina was cleansed with 20 mL of iodine solution. The cervix was grasped with colored forceps and gently retracted. The surgeon measured the uterine length using a uterine catheter (hysterometer) and inserted a dilator of the same length to protect surrounding tissues. After induction of anesthesia, endometrial and endocervical biopsies, along with polypectomies, were performed using a curette suction catheter (Unimar Inc. Wilton, Conn.). Figure 2 shows the timeline of disease progression in the course of endometritis with AUB and the stages of diagnosis.

## 3 Methods

**FIGURE 3:**
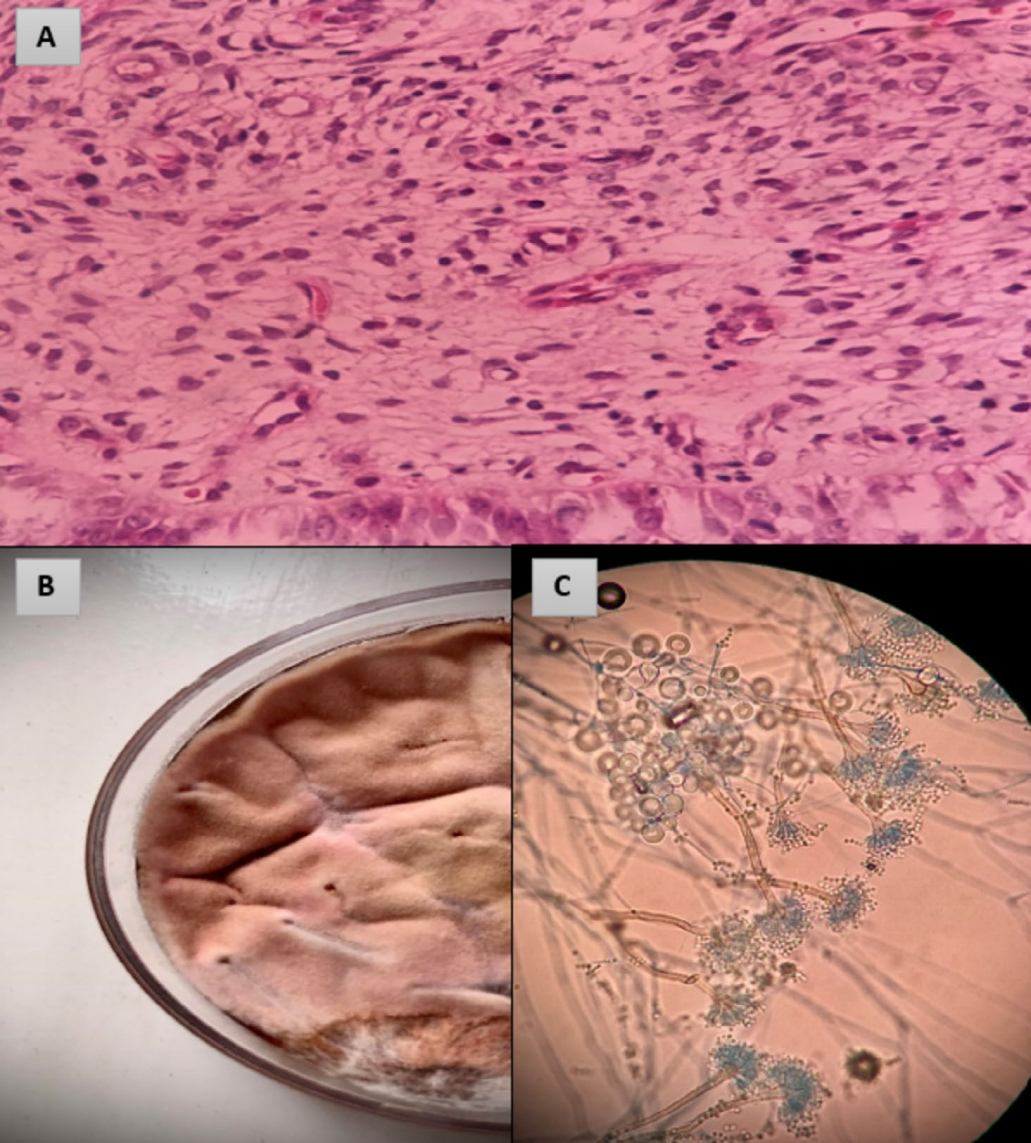
(A) Septate hyphae on endocervix tissue biopsy using H&E staining (B) *Aspergillus quadrilineatus* in sabouraud dextrose agar after 3 days growth on 37°C (C) Microscopic morphology of *A. quadrilineatus*, ×40 using lactophenol cotton blue.

The samples were sent to the laboratory for examination in a sterile container. Histopathological hematoxylin and eosin (H&E) staining demonstrated septate hyphae, resembling molds, which suspected the presence of fungal infections (Figure 3).

Fungal culture became positive 2 days after inoculating the samples on Sabouraud dextrose agar (Merck, Germany) plate, demonstrating growth of filamentous fungi with morphological features (conidiophore, vesicle, hull cell, and phialide) consistent with *Aspergillus* species.

To extract DNA using the grinder‐phenol‐chloroform method [13], mycelium pieces were initially crushed in a lysis buffer using an electric mill. The resulting supernatant was transferred to a new tube, to which an equal volume of phenol and chloroform was added. The mixture was then centrifuged. The supernatant was carefully transferred to another tube, and an equal volume of 2‐propanol, along with one‐third the volume of sodium acetate, was added at a temperature of 23°C. The solution was subsequently frozen in a −20°C freezer for 20 min. Following this, the sample was sonicated in the microwave for 10 min at a speed of 12,000 g. The sediment that formed at the bottom of the tube was clearly visible. To remove salts and residual alcohol, the sample was washed thoroughly with 70% ethanol. Finally, the resulting sediment was resuspended in 20 μL of double‐distilled water and stored at −20°C.

To conduct PCR, 5 μL of extracted DNA were combined with 12.5 μL of Master Mix and 0.4 μM primer. Sterile deionized distilled water was added to achieve a final reaction volume of 25 μL, which was subsequently placed in a thermal cycler. The temperature program was established with an initial denaturation step at 95°C for 5 min, followed by 35 cycles comprising a denaturation step at 94°C for 45 s, an annealing step at 55°C for 45 s, and an extension step at 72°C for 1 min. A final extension step was performed at 72°C for 6 min. To analyze the reaction results, 7 μL of the PCR product were mixed and subjected to electrophoresis on a 1.5% agarose gel in a tank containing TBE buffer (EDTA, boric acid, and Tris). The gel was visualized using a transilluminator, and images of the bands were captured for documentation.

Subsequently, the isolate was sequenced using the β‐tubulin gene as described previously [14]. The obtained sequences were compared with those in GenBank (www.ncbi.nlm.nih.gov).

In vitro antifungal susceptibility test of six antifungal drugs was performed according to the clinical laboratory standard institute (CLSI) guidelines M38‐A3 [15]. Stock solutions of drugs were prepared in dimethylsulfoxide (DMSO) (Sigma‐Aldrich, St. Louis, MO, USA) and then diluted in RPMI 1640 medium (Sigma‐Aldrich, St. Louis, MO, USA) buffered to pH 7.0 with morpholine‐propanesulfonic acid buffer (MOPS, Sigma‐Aldrich, St. Louis, MO, USA). Final concentrations ranged from 0.064–64 μg/mL for fluconazole and 5‐flucytosine (Pfizer, Groton, CT, USA), 0.016–16 μg/mL for itraconazole (Janssen, Beerse, Belgium), clotrimazole (Sigma, St. Louis, MO, USA), voriconazole (Sigma, St. Louis, MO, USA) and amphotericin B (Sigma, St. Louis, MO, USA). The minimum inhibitory concentration (MIC) for 50% inhibition of fungal growth for both azoles and amphotericin B (100%) were determined visually after incubating at 35°C for 48 h. *Candida parapsilosis* ATCC 22019, *Paecilomyces variotii* ATCC 22319were chosen as quality controls.

A comprehensive search of various databases “Web of Science”, “PubMed”, “Science Direct”, and “Scopus” was conducted to find relevant articles about fungal infections caused by *A. quadrilineatus*. The search was limited to published literature in English up to December 31, 2024.

## 4 Conclusion and Results

Macroscopic and microscopic fungal studies were performed, which led to tentative identification as *Aspergillus* species. The morphological characteristics of *Aspergillus quadrilineatus* include short, columnar and bilateral conidial heads. Additionally, conidiophore tips are brown, short, and smooth‐walled, while conidia are rough‐walled and spherical. (Figure 3). The isolate with accession number PQ594834 was identified as *A. quadrilineatus* , which showed 100% similarity with the type strains of that species (accession numbers OP265135.1 and KU866850.1). The MICs for voriconazole, itraconazole, amphotericin B, fluconazole, 5‐flucytosine and clotrimazole against *A. quadrilineatus* were 0.032, 0.25, 0.032, and 0.032 μg/mL. 4 and 0.5 μg/mL, respectively.

Following the diagnosis of the cause of the disease, fluconazole 150 mg capsules were prescribed for the patient at a dosage of one capsule every 72 h for a total of three doses. This treatment regimen appears to be an effective option for addressing the disease, in conjunction with curettage and debridement. During the patient's 6‐month follow‐up evaluation, the physician evaluated the patient's symptoms, performed a pelvic exam, and ordered additional diagnostic tests, including an ultrasound and a culture of vaginal secretions. The patient was instructed in appropriate hygiene practices and advised to report any new symptoms or worsening of existing symptoms. For the effective management of fungal endometritis, it is essential to adhere to appropriate hygiene protocols alongside medical treatment. These protocols encompass the maintenance of genital hygiene, the avoidance of gels, lotions, scented genital cleansers, and vaginal douches, as well as the use of intrauterine devices (IUDs) [16]. Additionally, the incorporation of probiotics into the diet is recommended. The findings from the negative vaginal sample culture, the normal ultrasound examination, and the patient's reported symptoms collectively confirmed a complete recovery.

According to the latest findings, a total of eight cases of *A. quadrilineatus* were found in the world, which were related to fungal infections, and the details are presented in Table 1. This case presents the first clinical example of endometrial aspergillosis caused by *A. quadrilineatus* in a patient presenting with AUB, highlighting the importance of this fungus in uterine infections. Curettage and debridement are suggested as effective treatment options for this disease. Due to the increasing prevalence of fungal infections caused by cryptic species and their potential resistance to standard antifungal agents, screening studies to assess the prevalence of these species are strongly recommended [17]. Furthermore, more comprehensive information on antifungal susceptibility is needed before recommending first‐line treatments for infection in high‐risk patients. The use of debridement in the management of fungal endometritis, especially in acute and severe cases, is favored for compelling reasons. Primarily, debridement exhibits the ability to directly penetrate the site of infection and eradicate the pathogen, an advantage not usually provided by oral or parenteral drugs that may not adequately reach the site of uterine infection. In addition, debridement shows better efficacy compared to systemic antifungal agents in preventing the spread of infection to nearby organs if it is combined with an oral antifungal drug such as fluconazole. In addition, the administration of some antifungal drugs may lead to adverse side effects, which are relatively less common in cases where debridement is used [18].

**TABLE 1:** Demographic characteristics, clinical data, and treatment profiles of reported cases due to *A. quadrilineatus* .

Case no.	Study	Year	Sex/age	Predisposing factor	Involved site	Antifungal therapy	Outcome
1	Polacheck et al. [14]	1992	F/28	ANLL	Sinusitis	AmB	Survived
2	Gugnani et al. [15]	2004	M/60	No	Nail	Itraconazole	Survived
3	Verweij et al. [16]	2008	M/10	CGD	Lung involvement	ND	ND
4	Sharma et al. [17]	2015	M/45	No	Nail	Terbinafine	Survived
5	Salah et al. [18]	2019	F/25	No	Nail	ND	Survived
6	Amirizad et al. [19]	2023	F/11	ALL	Cerebral	Caspofungin and AmB	Died
7	Zhang et al. [20]	2024	M/91	Type 2 diabetes, coronary heart disease and lower extremity arterial occlusive disease	Lung involvement	Voriconazole	Survived
8	Current study	2024	F/64	No	Endometer, uterus	Debriment and Fluconazole	Survived

Abbreviations: ALL, acute lymphoblastic leukemia; AmB, amphotericin B; ANLL, acute non‐lymphoblastic leukemia; CGD, chronic granulomatosis disease; ND, not determined.

## 5 Discussion

Endometrial infections are becoming increasingly prevalent among women, with conditions such as diabetes exacerbating their incidence [2]. Current case is derived from an unpublished study conducted by the authors of this article, which focused on women experiencing refractory AUB. The findings of the research indicate that there is no significant relationship between age and susceptibility to fungal endometritis, as observed in this study, and women beyond reproductive age are also at risk of contracting this infection.

In previous studies [19], the most frequently cited factors were *Candida* species, which have been identified in endometrial tissue in both humans and animals [20]. This prevalence may be associated with recurrent vulvovaginal candidiasis infections [2]. Conversely, the presence of aspergillosis in endometrial tissue has not been documented in humans. Nevertheless, given the numerous reports of *Aspergillus* species in Pap smear samples, the potential presence of *Aspergillus* in endometrial tissue warrants further consideration [12]. While *Aspergillus* hyphae are typically characterized by their acutely branched structures, distinguishing them from the hyphae forms of other fungi, such as *Pseudallescheria boydii*, *Fusarium* species, and *Candida* species, can prove challenging. The identification and confirmation of *Aspergillus* often necessitate microbiological isolation through culture, while histological diagnosis can be established upon the observation of *Aspergillus* fruiting bodies. Molecular techniques, including PCR, can be utilized to detect *Aspergillus* DNA. *A. quadrilineatus* belongs to the section *Nidulantes* and is phenotypically similar to *A. quadrilineatus* (formerly *Emericella quadrilineata*) is a soil fungus commonly isolated in tropical regions [21]. It has been identified as a causative agent of fungal sinusitis, onychomycosis, and invasive infections in patients with chronic granulomatous disease (CGD) and leukemia [22]. Notably, these two sibling species are morphologically identical but can be distinguished by molecular differences through PCR sequencing of the β‐tubulin gene. Notably, the patient under investigation exhibited neither immunodeficiency nor a known drug history [1]. In 50% of patients reported with *A. quadrilineatus*, underlying diseases have been identified. Notably, with the exception of one case that involved brain involvement, the rest showed predominantly respiratory system involvement. Conversely, in cases where there was no underlying disease, 75% of cases were localized to the nails [15], with only one documented case of fungal endometritis leading to a deeper organ infection. Historically, *Candida* species have been identified as the primary causative agents of endometrial infections; however, emerging evidence suggests that *Aspergillus* also has the potential to infect the ovary and uterus [2]. Following the confirmation of *Aspergillus* in any clinical sample, further investigation is warranted to exclude systemic involvement. In this case study, the patient was examined for vulvovaginal conditions, vaginal bleeding, abdominal pain, and fever for 4 months after sampling. The review of studies showed that the mean age of the patients was 36.70 years and the overall mortality rate was approximately 14.28%. The most prevalent infection was nail disorder (37.5%), lung infections (25%), followed by sinus and cerebral involvement (14.28%). Despite their close morphological and genetic relatedness, the two species exhibit significant differences in their in vitro susceptibility to AmB; however, triazoles demonstrate in vitro activity against both species. The antifungal drug sensitivity test conducted in this study showed that azole and AmB drugs showed strong antimicrobial activity against *A. quadrilineatus* , while as shown in the study by Zhang et al., it seems that 5‐Fluorocytosine shows weak antimicrobial activity against *Aspergillus* [23]. According to the latest studies, voriconazole, posaconazole, isavuconazole and echinocandins are used to treat infections caused by this species [21].

## Author Contributions

**Fatemeh Zahra Ranjbar Golafshani:** conceptualization, methodology, resources, writing – original draft, writing – review and editing. **Erfan Ghaffari Lashkenari:** data curation, investigation, software, writing – review and editing. **Soheila Abbaszadeh Godarzi:** data curation, formal analysis, validation, writing – original draft, writing – review and editing. **Firoozeh Kermani:** conceptualization, data curation, software, writing – original draft, writing – review and editing. **Saeid Mahdavi Omran:** project administration, resources, supervision, visualization, writing – original draft.

## Ethics Statement

Written informed consent was obtained from the patient before the commencement of this research. The study was conducted by Babol University of Medical Sciences in compliance with the ethical guidelines specified under code IR.MUBABOL.HRI.REC.1402.285.

## Consent

The authors affirm that human research participants provided informed consent for publication.

## Conflicts of Interest

The authors declare no conflicts of interest.

## Data Availability

The datasets used and/or analyzed during the current study are available from the corresponding author on reasonable request.
